# Maximum walking speed in multiple sclerosis assessed with visual perceptive computing

**DOI:** 10.1371/journal.pone.0189281

**Published:** 2017-12-15

**Authors:** Anuschka Grobelny, Janina R. Behrens, Sebastian Mertens, Karen Otte, Sebastian Mansow-Model, Theresa Krüger, Elona Gusho, Judith Bellmann-Strobl, Friedemann Paul, Alexander U. Brandt, Tanja Schmitz-Hübsch

**Affiliations:** 1 Charité – Universitätsmedizin Berlin, corporate member of Freie Universität Berlin, Humboldt-Universität zu Berlin, and Berlin Institute of Health, NeuroCure Cluster of Excellence, NeuroCure Clinical Research Center, Berlin, Germany; 2 Motognosis UG, Berlin, Germany; 3 Charité – Universitätsmedizin Berlin and Max Delbrück Center for Molecular Medicine, Experimental and Clinical Research Center, Berlin, Germany; 4 Charité – Universitätsmedizin Berlin, corporate member of Freie Universität Berlin, Humboldt-Universität zu Berlin, and Berlin Institute of Health, Department of Neurology, Berlin, Germany; Tokai University, JAPAN

## Abstract

**Background:**

Gait is often impaired in people with multiple sclerosis (PwMS), but detailed assessment of gait impairment in research and care remains challenging. In a previous pilot study we reported the feasibility of visual perceptive computing (VPC) for gait assessment in PwMS using the Short Maximum Speed Walk (SMSW), which assesses gait on recording distances confined to less than 4 meters.

**Objective:**

To investigate the equivalence of SMSW to rater-based timed 25ft. walk (T25FW) in a large cohort of PwMS, and to investigate the association of SMSW-derived gait parameters with clinical disability, as well as subjective and objective gait impairment, in order to validate the SMSW as a quick and objective measure of clinical relevance possibly superior to T25FW.

**Methods:**

95 PwMS and 60 healthy controls (HC) performed the SMSW using a VPC system with Microsoft Kinect. All participants received two immediate retests to establish test-retest-reliability. Both PwMS and HC performed the T25FW. PwMS were rated according to the Expanded Disability Status Scale (EDSS) and answered the 12-item Multiple Sclerosis Walking Scale (MSWS-12) as a measure of self-perceived walking impairment.

**Results:**

PwMS showed reduced average speed (p<0.001) and higher mediolateral deviation (p = 0.002) during SMSW than HC. Average speed was the most reliable SMSW parameter in PwMS and HC (intra-class correlation coefficient (ICC) in PwMS = 0.985, and in HC = 0.977). Average speed declined with age in PwMS and HC (r in PwMS = -0.648, and in HC = -0.452, both p<0.001). Correlation of SMSW average speed and T25FW speed was high in both groups (r in PwMS = 0.783, and in HC = 0.747, both p<0.001) and mean difference (0.0013 m/s) between methods was below smallest detectable change. Average speed correlated well with both clinical disability based on EDSS (r = -0.586, p<0.001) and self-perceived walking impairment based on MSWS-12 (r = -0.546, p<0.001).

**Conclusion:**

VPC-assessed walking parameters during SMSW can reliably detect gait disturbance in PwMS over very short distance. Specifically, maximum gait speed can be obtained with high accuracy in this simple test set-up. Cross-sectional associations with disability and self-perceived walking impairment support clinical relevance. Given its objectivity in a simple test set-up, SMSW is superior to T25FW.

## Introduction

Multiple sclerosis (MS) results in demyelination as well as axonal and neuronal loss. [1] People with MS (PwMS) worry most about their walking abilities[2] and the majority of PwMS has ambulatory deficits, e.g. slower walking speed.[3] Walking problems are strongly associated with the risk of falling,[4] increased healthcare utilization and reduced quality of life.[5] The relevance of gait speed as a global measure of functional capacity and predictor for functional decline in various conditions led to its designation as the “sixth vital sign”.[6]

Gait disorders in PwMS are commonly assessed as maximum free walking distance in the Expanded Disability Status Scale (EDSS)[7] or as decline in maximum walking speed in timed walks, e.g. the Timed 25-Foot Walk (T25FW) contained in the multiple sclerosis functional composite (MSFC).[8] Compared to EDSS, the T25FW is a more reliable[8,9] and valid measure.[10] It has been used as a primary[11] or secondary outcome in multiple MS trials, reported as either performance time or speed.[12]

Visual perceptive computing (VPC) with Microsoft’s Kinect has been proposed as a feasible and inexpensive method to quantify gait[13–15] and postural control.[15–17] In a pilot study, we introduced the Short Maximum Speed Walk (SMSW)[13] in a small group of PwMS and healthy controls (HC). Based on the promising results, the pilot study was extended.

We here report results of this cross-sectional study extension. It was our main objective to validate the SMSW as a rater-independent, objective method to quantify gait, that can be applied in both research and care and that is possibly superior to conventional stopwatch testing (T25FW). We report on test-retest-reliability as well as demographic confounding factors of a set of five parameters derived from SMSW. We show the clinical relevance of SMSW by between-group comparison and association with clinical disability and self-perceived impairment in everyday-locomotor activities.

## Material and methods

### Patients and controls

This cross-sectional observational study included a convenience sample of 95 PwMS (EDSS ≤ 6.0) according to McDonald Criteria 2010[18] and 60 age-, sex- and BMI-matched HC. Subjects were enrolled from a neuroimmunology outpatient service at a university referral center from September 2013 to April 2014. The study was approved by the local ethics committee of the Charité –Universitätsmedizin Berlin (EA1/225/12) in conformity with the Declaration of Helsinki in its currently applicable form. All participants gave written informed consent. Pilot data from a subset of study participants on SMSW feasibility and applicability have been previously reported.[13] Five PwMS were excluded due to motor impairment other than MS. VPC quality control failed in another seven PwMS and three HC, and data from these participants were thus excluded prior to analysis (see below), leading to a total of 83 included PwMS and 57 HC (Table 1).

**Table 1:** Cohort overview.

		HC	PwMS	Statistic	p
**Subjects**	*n*	57	83		
	*RRMS*	.	*72 (86*.*7%)*		
	*SPMS*	.	*8 (9*.*6%)*		
	*PPMS*	.	*3 (3*.*6%)*		
**Sex**	Male	24 (42.1%)	34 (41.0%)	Chi^2^ = 0.018	0.893
	Female	33 (57.9%)	49 (59.0%)		
**Age (years)**	Mean ± SD Age range (years)	40.7 ± 14.2 18–66	43.0 ± 10.6 22–67	T = -1.117*	0.266
**BMI**	Mean ± SD	24.8 ± 4.1	25.5 ± 4.9	T = −0.879°	0.381
**Height (m)**	Mean ± SD	1.72 ± 0.07	1.75 ± 0.10	T = -1.827*	0.070
**Weight (kg)**	Mean ± SD	73.7 ± 15.1	77.8 ± 15.8	T = -1.546*	0.125
**T25FW speed (m/s)**	Mean ± SD	1.85 ± 0.28	1.65 ± 0.34	T = 3.870°	<0.001
**MSWS-12**	Mean ± SD	.	25.4 ± 24.1	T = -7.936*	<0.001
**EDSS**	Median (Min—Max)	.	2.8 (0.0–6.0)		

**Abbreviations:** HC: healthy controls. PwMS: people with multiple sclerosis. RRMS: relapsing-remitting multiple sclerosis. SPMS: secondary progressive multiple sclerosis. PPMS: primary progressive multiple sclerosis. SD: standard deviation. BMI: Body Mass Index. T25FW: Timed 25-Foot Walk Test. MSWS-12: walking scale 12-item. EDSS: Expanded Disability Status Scale.

*Student’s t-test.

°Welch’s t-test.

### Clinical assessment

Subjects performed VPC testing (see below) and clinical examinations in one session: All subjects completed the T25FW with start from standing as part of the Multiple Sclerosis Functional Composite (MSFC).[8] To enable direct comparison with SMSW average speed, T25FW performance time was converted to speed (m/s) as 7.62 m / *T*25*FW* (*s*). The self-reported impact of MS on walking ability was documented with the 12-item Multiple Sclerosis Walking Scale (MSWS-12) applied as an interview. The MSWS-12 sum score of the five-step scoring of each item was transformed to a range of 0–100 as follows: $(\frac{sum-12}{48})\;\times \;100$, with 0 meaning full walking ability and 100 meaning complete loss of walking ability.[19] PwMS were additionally scored based on the Expanded Disability Status Scale (EDSS) by trained clinical investigators under supervision of a board certified neurologist.[7] Eighty-one PwMS and all HC performed the T25FW. 82 PwMS and answered the MSWS-12 questionnaire.

### Visual perceptive computing

**Fig 1:**
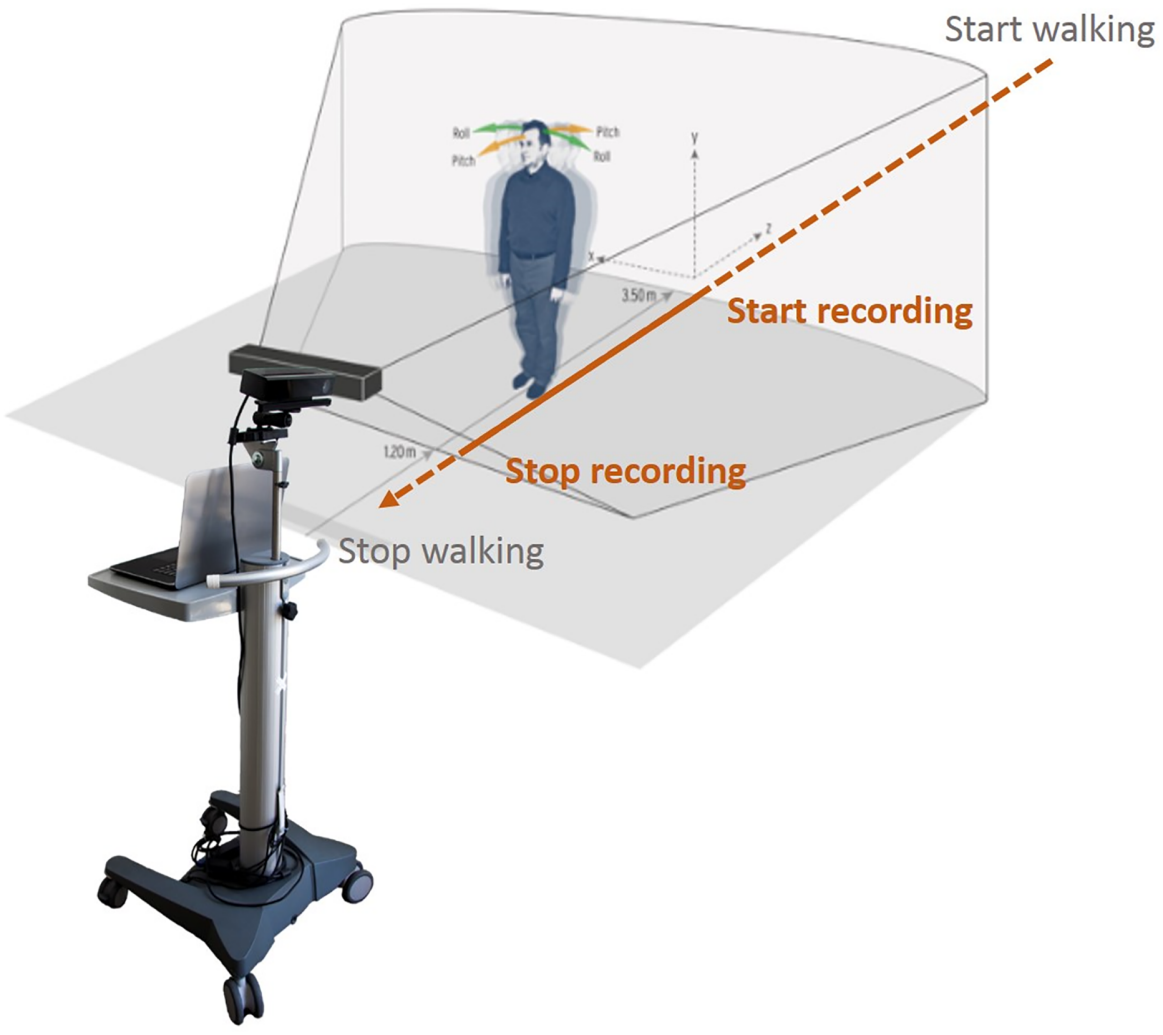
Test set-up.

VPC-based motor assessment was performed using a Motognosis Labs System V1.0 (Motognosis, Berlin, Germany) equipped with a Kinect V1 for Windows sensor and Kinect Software Development Kit (SDK) versions 1.7 and 1.8 (Microsoft, Redmond, WA, USA). The SDK uses the reflections of an array of infrared signals to detect subject position and projects an artificial skeleton with 20 artificial joints into the body shape. Tests were performed in an evenly lit physician’s office with regular footwear. After oral operator instructions, audio signals indicated test start and end of recording. Construct validity of Kinect 1 and 2 systems for gait assessment has been previously reported by others and us.[14,15,17] Subjects started from a 5 m distance and were instructed to walk as fast as possible towards the camera. Recording started automatically when the subject entered the recording space at approximately 3.5 m and ended at about 1.5m distance to the camera (Fig 1).

The coordinates of the “hip center joint” were used to generate SMSW output. We analyzed five parameters as reported previously (Table 2).[13]

**Table 2:** SMSW parameter overview.

SMSW parameter	Definition
**Average speed (m/s)**	total distance travelled in anterior-posterior direction per recording time
**Speed deviation (m/s)**	standard deviation of speed between subsequent pairs of frames
**Mediolateral deviation (cm)**	mediolateral standard deviation of the aligned anterior-posterior-vector
**Vertical deviation (cm)**	vertical standard deviations of the aligned anterior-posterior-vector
**3D deviation (cm**^**2**^**)**	combined expression of all directional variability of movement

To analyze test-retest-reliability, all subjects performed three immediate test repetitions. All VPC joint time series were visually inspected for data quality and datasets were excluded from analysis in cases of obvious mismatch of Kinect artificial joints with anatomical landmarks (failure A) or when the execution time was less than 2 s and therefore considered too short to reliably analyze SMSW parameters (failure B). In these cases, all three trials of the subject were discarded. Of the remaining datasets, data plots of all three test repetitions were inspected and values outside a range of three standard deviations of group means in only single trials of a subject considered implausible (failure C) and therefore excluded. All subjects were able to perform VPC testing. Three HC and four PwMS were excluded due to failure A and one PwMS was excluded due to both failure A and B. Two PwMS were excluded due to failure C in speed deviation.

### Statistical analysis

Data were analyzed for normality by visual inspection of histograms and calculation of skewness and kurtosis. A skewness or kurtosis outside +/-1.5 was considered evidence of a non-normal distribution. Based on these analyses, speed deviation in HC and 3D deviation in PwMS showed a non-normal distribution, whereas all other data were normally distributed. To account for potential distribution effects in speed deviation and 3D deviation, we confirmed significance levels using non-parametric testing (Mann-Whitney U test for group comparisons and Spearman’s Rho for correlation analyses), but retained parametric effect sizes and p-values in the presented results to allow comparability between parameters. The significance levels of results were confirmed for all analyses. Test-retest-reliability was analyzed using intra-class correlation coefficients (ICC) based on an absolute agreement two-way mixed-effects model.[20] Interpreting ICC values, reliability was classified as poor (less than 0.5), moderate (0.5–0.75), good (0.75–0.9) and excellent (more than 0.9).[20] Standard error of measurement was calculated as *SEM* = *SD within group from* 1*st test* × √(1 − *ICC*).[21] The SEM was additionally expressed as proportion of the mean (SEM%). We further computed the smallest real difference as *SRD* = 1.96 × *SEM* × √2 Actual score differences between two assessments can be assumed as true signal with 95% confidence, when they are greater than the SRD.[21] In order to investigate, if there was a directional effect e.g. due to learning, fatiguing or disengagement between the three test repetitions (r1, r2, r3), we conducted a one-way repeated measures ANOVA separately for HC and PwMS with subsequent pairwise comparisons.

The average of all three SMSW trials was then used for all further analyses. Possible confounding influences of subject’s age, sex, height and weight were analyzed by multivariate linear regressions per variable (enter method). To answer the main objective of the study, we chose average speed of our test paradigm for comparison to T25FW speed. We performed Pearson correlation of both measures of maximum walking speed and visualized their agreement as Bland-Altman-plot. All parameters were compared between PwMS and HC using Student‘s t-test, when equal variances were assumed based on Levene‘s test of equal variances, otherwise Welch‘s t-test was used. Spearman correlations were used to analyze associations with EDSS, and Pearson correlation to analyze associations with MSWS-12. Statistical analysis was performed with SPSS, version 23 (IBM, Armonk, NY, USA). All tests were two-tailed, significance was assumed when p<0.05, unless otherwise noted.

## Results

PwMS had slower average speed and greater mediolateral deviation than HC. There was a trend towards greater speed deviation in PwMS, while both groups did not differ in vertical and 3D deviation (Table 3).

**Table 3:** Group differences between PwMS and HC.

SMSW parameter	PwMS	HC	Statistic		
	Mean ± SD	Mean ± SD	Difference	T	p*
**Average speed (m/s)**	1.66 ± 0.30	1.83 ± 0.26	0.17	3.526°	**0.001**
**Mediolateral deviation (cm)**	1.33 ± 0.32	1.15 ± 0.32	-0.18	-3.200°	**0.002**
**Vertical deviation (cm)**	1.72 ± 0.43	1.78 ± 0.48	0.06	0.794°	0.429
**Speed deviation (m/s)**	0.20 ± 0.03	0.19 ± 0.03	-0.01	-1.770°	0.079^§^
**3D deflection deviation (cm**^**2**^**)**	5.02 ± 1.84	4.78 ± 2.04	-0.24	-0.700°	0.485

**Abbreviations:** PwMS: people with multiple sclerosis. HC: healthy control. SMSW: Short Maximum Speed Walk. SD: standard deviation.

°Welch’s t-test.

*p-values less than 0.01 were deemed significant after Bonferroni correction (indicated in bold)

^§^ non-significance after Bonferroni correction of this non-normally distributed parameter was confirmed with Mann-Whitney U test (p = 0.045)

### Test-retest-reliability and smallest real difference

Next, we established SEM and SRD in PwMS and HC. For this, we performed ICC analysis, from which we then calculated SEM and SRD. Test-retest-reliability reached significance for all SMSW parameters in both HC and PwMS (all p<0.001). Average speed proved to be the most reliable parameter in HC and PwMS with excellent reliability based on the ICC and its 95% confidence intervals.[20] Speed deviation was the least reliable parameter in HC and mediolateral deviation showed the lowest ICC in PwMS (Table 3). Accordingly, the SEM, when expressed as the percentage of group mean, was minimal for average speed in both groups (PwMS/HC both 2.2%) but higher for all other parameters. Only for average speed, the observed group difference between HC and PwMS exceeded the SRD (Table 4).

**Table 4:** Test-retest-reliability and smallest real difference.

SMSW parameter	PwMS					HC				
	ICC	95% CI	SEM	SEM%	SRD	ICC	95% CI	SEM	SEM%	SRD
**Average speed (m/s)**	0.985	0.979–0.990	0.04	2.4%	0.10	0.977	0.965–0.986	0.04	2.2%	0.11
**Mediolateral deviation (cm)**	0.507	0.290–0.666	0.34	25.6%	0.62	0.774	0.650–0.859	0.16	13.9%	0.42
**Vertical deviation (cm)**	0.920	0.885–0.946	0.14	8.1%	0.34	0.933	0.896–0.958	0.13	7.3%	0.34
**Speed deviation (m/s)**	0.771	0.670–0.845	0.02	8.7%	0.05	0.693	0.523–0.809	0.03	15.4%	0.06
**3D deflection deviation (cm**^**2**^**)**	0.793	0.702–0.860	1.11	22.1%	2.32	0.906	0.854–0.941	0.62	13.0%	1.73

**Abbreviations:** PwMS: people with multiple sclerosis. HC: healthy control. SMSW: Short Maximum Speed Walk. SD: standard deviation. ICC: intra-class correlation coefficient. CI: confidence interval. SEM: standard error of measurement. SRD: smallest real difference.

For HC, there was no significant directional change over the three repetitions (p = 0.077). For PwMS there was a significant increase in speed over the three repetitions (p = 0.046). Pairwise comparisons showed that this difference was based on a significant increase of speed between r1 and r2 (mean difference 0.23 m/s, p = 0.041), whereas PwMS became slower from r2 to r3 (-0.07 m/sec, p = 1.000). When comparing only r1 to r3, the speed increase was not significant anymore (0.16 m/s, p = 0.425). Overall, %SEM was small in both groups and it thus seems well justified to use mean of three trials.

### Association with age, sex, height and weight

We then investigated potentially confounding demographic factors for SMSW measurements both in HC and PwMS (Table 3). In HC, age was the only influencing factor for average speed (p = 0.001) and the main factor for T25FW speed (p = 0.001). In HC, T25FW speed was also influenced by height (p = 0.045), but this effect did not reach significance for average speed in HC nor for both measures of maximum speed in PwMS. This may be interpreted as a mild effect of height on maximum gait speed apart from the larger age effect especially in HC. Models for mediolateral and 3D deviation in HC were determined by sex (p = 0.010 and = 0.014) with more mediolateral deviation in males (p = 0.009). In PwMS, age was the main determinant in all models (all p<0.001). In contrast to HC, sex had no effect on gait parameters (Table 5).

**Table 5:** Demographic confounders in HC (A) and PwMS (B).

	Adj. R^2^	Model Sig.	Age Beta	Sex Beta	Height Beta	Weight Beta
**A**						
**Average speed (m/s)**	**0.210**	**0.002**	-0.435 p = 0.001	0.175	0.287	0.090
**Mediolateral deviation (cm)**	**0.150**	**0.014**	0.097	-0.498 p = 0.010	-0.327	0.135
**Vertical deviation (cm)**	**0.074**	**0.093**	-0.063	-0.285	0.253	-0.310
**Speed deviation (m/s)**	**-0.053**	**0.877**	-0.035	-0.056	-0.188	0.010
**3D deviation (cm**^**2**^**)**	**0.123**	**0.028**	0.006	-0.481 p = 0.014	0.091	-0.217
**T25FW speed (m/s)**	**0.251**	**0.001**	-0.431 p = 0.001	0.016	0.348 p = 0.045	-0.127
**B**						
**Average speed (m/s)**	**0.434**	**<0.001**	-0.586 p<0.001	-0.034	0.205	-0.123
**Mediolateral deviation (cm)**	**0.154**	**0.002**	0.422 p<0.001	-0.152	-0.128	0.124
**Vertical deviation (cm)**	**0.205**	**<0.001**	-0.241 p = 0.022	-0.245	0.171	-0.150
**Speed deviation (m/s)**	**0.031**	**0.169**	0.286	0.009	0.085	0.007
**3D deviation (cm**^**2**^**)**	**0.049**	**0.095**	0.001	-0.253	0.091	-0.095
**T25FW speed (m/s)**	**0.357**	**<0.001**	-0.536 p<0.001	0.058	0.253	-0.190

Analyzed by multifactorial regression models. Model significance is reported along with each factor’s standardized beta coefficient; shaded cells indicate p<0.05.

**Fig 2:**
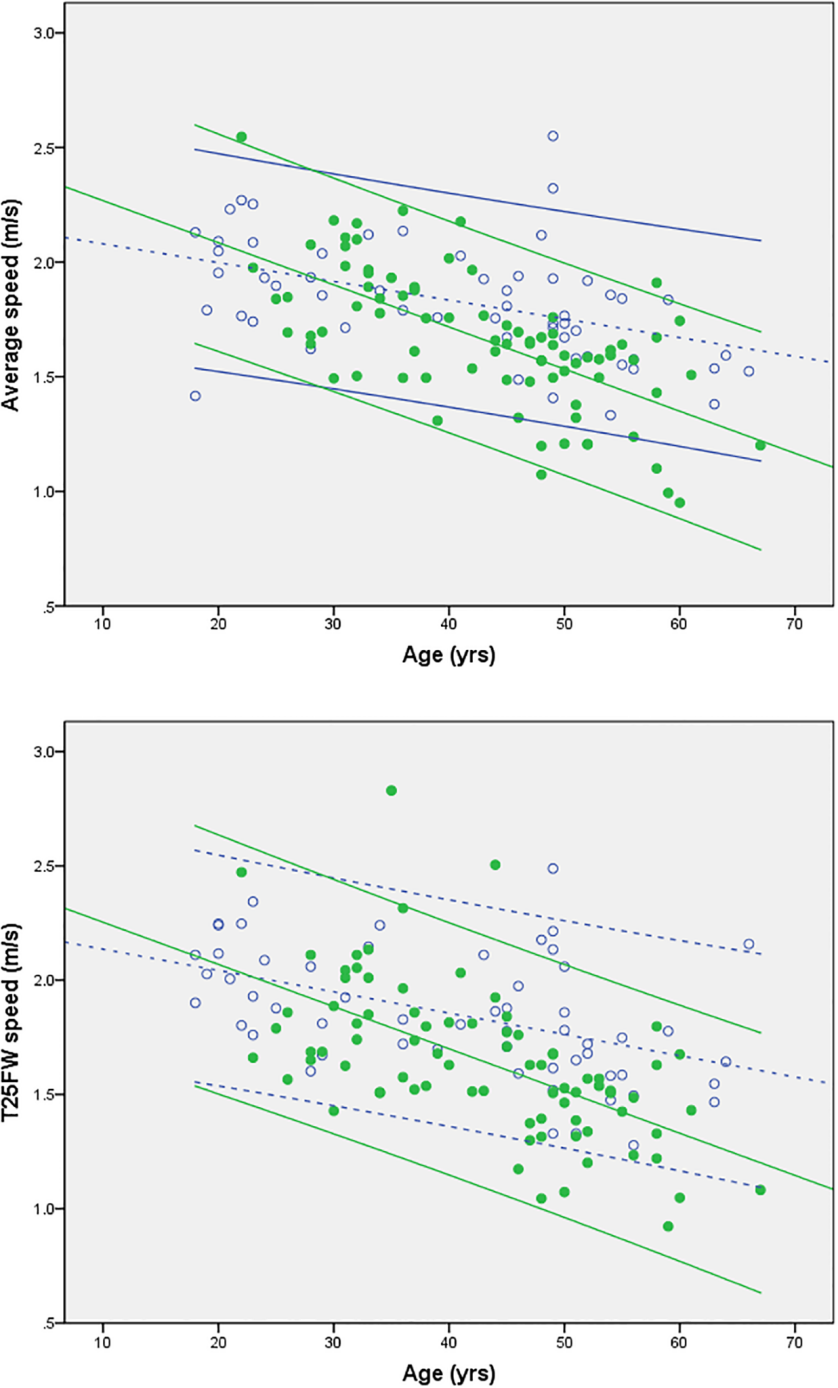
Univariate regression of maximum walking speed assessed with SMSW (average speed (A)) or T25FW (B) and with age as factor in HC (open circles, dashed lines) and PwMS (filled circles, continuous lines). Regression lines are given along with their 95% confidence intervals.

In both groups, T25FW (r in PwMS = -0.585, and in HC = -0.477, both p<0.001) and SMSW average speed (r in PwMS = -0.648, and in HC = -0.452, both p<0.001) showed a linear decline with age. This effect seems even more pronounced in PwMS (Fig 2).

Univariate regression analysis per group for the effect of age yielded the following equations to predict SMSW average speed:

$$\text{In}\;\text{HC}\;({\text{R}}^{2}=0.210):\;mean\;maximum\;speed\;\left(\frac{m}{s}\right)\;=\;-0.008\;\times \;age\;(years)\;+\;2.161.$$

$$\text{In}\;\text{PwMS}\;({\text{R}}^{2}=0.434):\;mean\;maximum\;speed\;\left(\frac{m}{s}\right)\;=\;-0.018\;\times \;age\;(years)\;+\;2.452.$$

### Association with clinical disability and self-perceived walking impairment

PwMS were characterized by slower T25FW speed than HC, and PwMS perceived their walking ability as compromised (Table 1). Reduced SMSW average speed and more mediolateral deviation correlated well with worse self-perceived walking impairment based on MSWS-12 (r = -0.546 and = 0.526, both p<0.001). PwMS with higher scores on MSWS-12 also showed more speed deviation (r = 0.245, p = 0.027), though this r-value indicated very weak correlation. Correlation analyses of SMSW parameters with EDSS scores in PwMS demonstrated slower average speed with higher disability (r = -0.586, p<0.001). Correlation analyses of mediolateral and speed deviation with EDSS were less robust (r = 0.373 and = 0.309, p = 0.001 and = 0.005). As expected from between-group comparison, vertical and 3D deviation did not show any relation to self-perceived walking impairment based on MSWS-12 or clinical disability based on EDSS. Correlations results with selected EDSS functional system (FS) scores indicate a similar relation of average speed to both pyramidal and cerebellar FS, while mediolateral and speed deviation are specifically related to the cerebellar FS (S1 Table).

### Equivalence of speed by SMSW and T25FW

**Fig 3:**
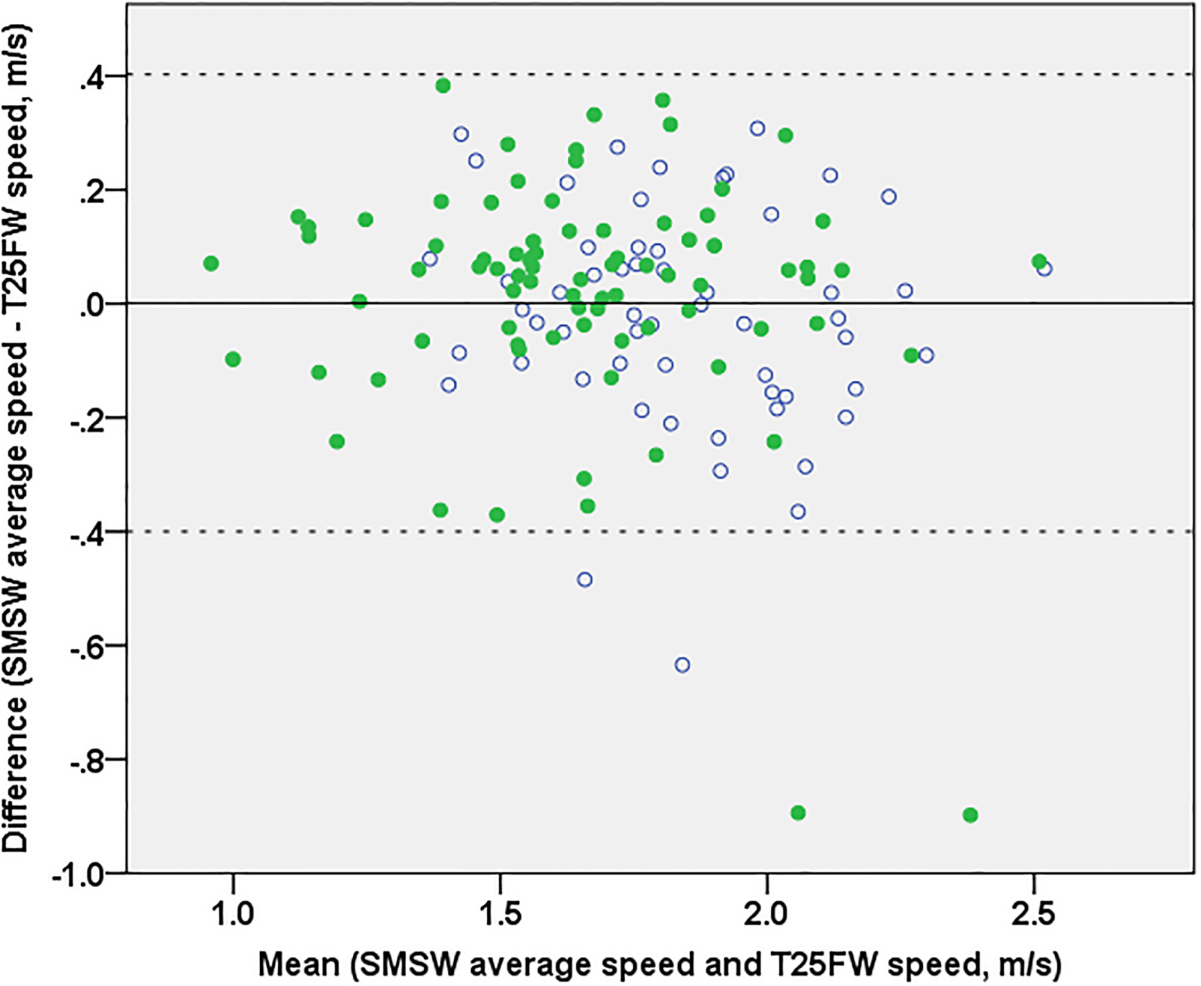
Bland-Altman plot of the differences between SMSW average speed and T25FW speed. Mean difference (solid line) and limits of confidence (dashed lines) refer to the whole dataset. For better interpretation, HC are rendered as open circles and PwMS as filled circles. In two HC and two PwMS, the difference between both maximum speeds was outside the limits of agreement. All four showed an overestimation of T25FW versus SMSW average speed but did not have any other specific feature in common.

At group level, average speed did not differ whether assessed with SMSW or T25FW neither in HC nor PwMS with a mean difference between methods of 0.0013 ± 0.2046 m/s when all subjects were pooled. Further, results for average speed from both tests were highly correlated in both groups (HC r = 0.747, p<0.001, PwMS r = 0.783, p<0.001). When data of PwMS and HC were pooled, the limits of agreement spanned from -0.3998 to 0.4024 without evidence of dependence on absolute speed values or group differences (Fig 3).

Expected from these results, average speed from both tests showed very similar correlations with the other VPC parameters in both groups (S2 Table). T25FW correlations with self-perceived gait impairment according to MSWS-12 (r = -0.456, p<0.001) and disability according to EDSS (r = -0.517, p<0.001) were somewhat lower in comparison to SMSW average speed (see above).

## Discussion

We here report data on marker-less VPC-based gait assessment using a one-camera protocol and customized software in a large cohort of PwMS and HC.

Clinical differences between groups were reflected in slower average speed and higher mediolateral deviation in PwMS. More mediolateral trunk movement during walking was also reported in 31 PwMS with normal walking speed using inertial-sensor based gait analysis.[22] Thus, reduced speed may in part be attributed to impaired dynamic balance during locomotion, which requires step-to-step sensorimotor feedback for mediolateral stability.[23] The specific relation of mediolateral excursion to cerebellar FS in our data point to this parameter as an indicator of cerebellar gait disturbance. As a limitation, test-retest-reliability for mediolateral deviation does not support its potential for individual disease monitoring. It should be noted, that mediolateral excursion during locomotion follows a physiological temporo-spatial pattern throughout one stride. Due to very short recording distances of our testing paradigm, only between one and two strides are recorded per trial, which may induce variability when only means over recording period are reported. Further study will explore, whether normalizing mediolateral excursion to stride may reduce variability and also sex dependency in HC and thus increase repeatability of this measure. We further aim to explore the potential of additional parameters of trunk and head stabilization during gait for use in MS, as changes in trunk movement have been suggested to occur early in the disease course.[22]

Excellent reliability was seen for average speed with a SEM of only 0.04 m/s (2% of mean) in both groups, which is well below the variability within groups (0.26 m/s) and the observed between-group difference (0.17 m/s). This was expected from ample evidence regarding reliability of timed walking tests.[24,25] The SRD of 0.11 m/s is very similar or even lower than those reported for both self-selected or maximum speed[6] and suggests that gait speed is most suited to track individual changes. VPC does not seem to add variability to gait speed assessment compared to other validated methods[14,15] or stop watch testing as shown in this study. However, immediate retest does not account for day-to-day variability of performance[9] that may even be a greater issue with impaired mobility.[26] The SRD based on between-session reliability, generally expected to be higher than within-session,[9] may be considered more appropriate to interpret individual changes and should be determined in future studies.

In both HC and PwMS average speed declined linearly with age. Age effects on maximum walking speed[24,27] and mean daily walking speed[28] have been reported with estimates of yearly decline between 0.004 and 0.016 m/s in HC. The steeper slope in PwMS seen here may be interpreted as a disease-related decline adding up to the physiological decline seen with ageing. Despite this, T25FW speed is usually reported without reference to subject’s age,[29] which may suffice for the observation of short term treatment effects. To distinguish slowed from normal walking speed on an individual level, however, we recommend using age-matched reference values. Similarly, maximum walking speed is usually rendered without scaling for body stature, despite some effect of stature seen here and reported elsewhere.[24]

The average speed of 1.83 m/s in our HC is within the limits of SRD compared to results from several studies[30,31] but lower than >2.1 m/s reported by others.[24] This applies to both methods of assessment and may be caused by the wording of patient instruction[32] among other effects of test setup,[27] whereas walking distance seems of less importance.[6] Despite dynamic start of SMSW and start from standing in T25FW used here, the mean difference in maximum speed is only 0.0013 m/s which indicates that maximum walking speed can be reliably assessed by SMSW. Rater-independent automated match of recording time to actual recording distance in SMSW may be essential for test precision at very short distances. In contrast, single overestimations of speed by T25FW may be explained by incorrect manual start or stop, though an effect of different test environment in single cases is not precluded. As the limits of agreement between SMSW and T25FW speed are larger than the SRD for average speed, follow-up should preferably use identical methods. However, in terms of sensitivity or predictive power, we consider both tests interchangeable.

The system used here generates output immediately without further pre-processing by the user and can be applied with minimal training. In confirmation of a pilot trial,[13] SMSW proved feasible in all participants from asymptomatic to moderate gait impairment (EDSS 0–6). After inspection of all individual assessments of this study, very few had to be excluded for reasons that can be resolved by either investigator instruction (failure A) or automated failure detection with prompting to repeat the trial (failure B and C). This underlines that proper test instructions remain important even when using technical motor assessment to enhance objectivity.[32] Despite high test-retest-reliability, we therefore recommend at least two SMSW repetitions to enable the detection of single implausible values with reference to population as well as re-test variance reported in this study.

In summary, SMSW is a valid, automated assessment of walking speed that is easy to handle. It is applicable in a broad range of patients, even in higher disabled people who are not able to walk 25 ft anymore. It reflects disability as well as subjective gait impairment and yields parameters of potential interest other than walking speed. We therefore propose VPC as a means to reliably perform gait testing in PwMS and consider SMSW superior to more time-consuming and rater-dependent clinical routine measures like EDSS or T25FW.

## Supporting information

S1 Table Correlations results of VPC parameters with selected EDSS FS scores. (DOCX)

S2 Table Correlations of average speed from SMSW and T25FW with the other VPC parameters in HC and PwMS. (DOCX)
