# Supplementary material for: Maximum walking speed in multiple sclerosis assessed with visual perceptive computing
Source: PLoS One. 2017 Dec 15;12(12):e0189281. doi: 10.1371/journal.pone.0189281 (PMC5731685; doi:10.1371/journal.pone.0189281)
Supplement: S1 Table — (DOCX) [file pone.0189281.s001.docx]

|  | Average speed (m/s) | Mediolateral deviaton (cm) | Vertical deviation (cm) | Speed deviation (m/s) | 3D deviation (cm²) |
| --- | --- | --- | --- | --- | --- |
| Pyramidal FS | r=-0.432  p<0.001 | r = 0.240  p = 0.030 | r=-0.092  p=0.421 | r=0.154  p=0.168 | r=0.066  p=0.553 |
| Cerebellar FS | r=-0.463  p<0.001 | r=0.388  p < 0.001 | r=-0.103  p=0.355 | r=0.295  p=0.007 | r=0.096  p=0.389 |
| Sensory FS | r=-0.278  p=0.011 | r=0.180  p=0.105 | r=-0.165  p=0.138 | r = 0.161  p=0.149 | r=-0.009  p=0.933 |
| Ambulation Score | r=-0.434  p<0.001 | r=0.351  p=0.001 | r=-0.005  p=0.962 | r = 0.192  p=0.084 | r=0.211  p=0.058 |
