# Supplementary material for: Maximum walking speed in multiple sclerosis assessed with visual perceptive computing
Source: PLoS One. 2017 Dec 15;12(12):e0189281. doi: 10.1371/journal.pone.0189281 (PMC5731685; doi:10.1371/journal.pone.0189281)
Supplement: S2 Table — (DOCX) [file pone.0189281.s002.docx]

| HC | Average speed (m/s) | Mediolateral deviaton (cm) | Vertical deviation (cm) | Speed deviation (m/s) | 3D deviation (cm²) | T25FW speed (m/s) |
| --- | --- | --- | --- | --- | --- | --- |
| Average speed (m/s) | . | T=-0.373  p=0.004 | T=0.104  p=0.439 | T=0.206  p=0.124 | T=-0.077  p=0.569 | T=0.747  p<0.001 |
| Mediolateral deviaton (cm) | T=-0.373  p=0.004 | . | T=0.147  p=0.274 | T=-0.064  p=0.636 | T=0.548  p<0.001 | T=0.350  p=0.008 |
| Vertical deviation (cm) | T=0.104  p=0.439 | T=0.147  p=0.274 | . | T=0.021  p=0.878 | T=0.895  p<0.001 | T=-0.040  p=0.770 |
| Speed deviation (m/s) | T=0.206  p=0.124 | T=-0.064  p=0.636 | T=0.021  p=0.878 | . | T=0.002  p=0.991 | T=0.102  p=0.452 |
| 3D deviation (cm²) | T=-0.077  p=0.569 | T=0.548  p<0.001 | T=0.895  p<0.001 | T=0.002  p=0.991 | . | T=-0.156  p=0.246 |
| T25FW speed (m/s) | T=0.747  p<0.001 | T=0.350  p=0.008 | T=-0.040  p=0.770 | T=0.102  p=0.452 | T=-0.156  p=0.246 | . |

| PwMS | Average speed (m/s) | Mediolateral deviaton (cm) | Vertical deviation (cm) | Speed deviation (m/s) | 3D deviation (cm²) | T25FW speed (m/s) |
| --- | --- | --- | --- | --- | --- | --- |
| Average speed (m/s) | . | T=-0.478  p<0.001 | T=0.346  p<0.001 | T=-0.259  p=0.018 | T=0.067  p=0.545 | T=0.783  p<0.001 |
| Mediolateral deviaton (cm) | T=-0.478  p<0.001 | . | T=-0.044  p=0.696 | T=0.280  p=0.010 | T=0.489  p<0.001 | T=-0.489  p<0.001 |
| Vertical deviation (cm) | T=0.346  p<0.001 | T=-0.044  p=0.696 | . | T=0.099  p=0.374 | T=0.830  p<0.001 | T=0.255  p=0.022 |
| Speed deviation (m/s) | T=-0.259  p=0.018 | T=0.280  p=0.010 | T=0.099  p=0.374 | . | T=0.232  p=0.035 | T=-0.208  p=0.063 |
| 3D deviation (cm²) | T=0.067  p=0.545 | T=0.489  p<0.001 | T=0.830  p<0.001 | T=0.232  p=0.035 | . | T=-0.014  p=0.900 |
| T25FW speed (m/s) | T=0.783  p<0.001 | T=-0.489  p<0.001 | T=0.255  p=0.022 | T=-0.208  p=0.063 | T=-0.014  p=0.900 | . |

Shaded cells indicate p<0.05.
